# High Through-Put Sequencing of the *Parhyale hawaiensis* mRNAs and microRNAs to Aid Comparative Developmental Studies

**DOI:** 10.1371/journal.pone.0033784

**Published:** 2012-03-20

**Authors:** Martin J. Blythe, Sunir Malla, Richard Everall, Yu-huan Shih, Virginie Lemay, Joanna Moreton, Raymond Wilson, A. Aziz Aboobaker

**Affiliations:** 1 Deep Seq, Faculty of Medicine and Health Sciences, Queen's Medical Centre, University of Nottingham, Nottingham, United Kingdom; 2 Evolutionary Developmental Biology Laboratory, Centre for Genetics and Genomics, Queen's Medical Centre, University of Nottingham, Nottingham, United Kingdom; Auburn University, United States of America

## Abstract

Understanding the genetic and evolutionary basis of animal morphological diversity will require comparative developmental studies that use new model organisms. This necessitates development of tools for the study of genetics and also the generation of sequence information of the organism to be studied. The development of next generation sequencing technology has enabled quick and cost effective generation of sequence information. *Parhyale hawaiensis* has emerged as a model organism of choice due to the development of advanced molecular tools, thus *P. hawaiensis* genetic information will help drive functional studies in this organism.

Here we present a transcriptome and miRNA collection generated using next generation sequencing platforms. We generated approximately 1.7 million reads from a *P. hawaiensis* cDNA library constructed from embryos up to the germ band stage. These reads were assembled into a dataset comprising 163,501 transcripts.

Using the combined annotation of Annot8r and pfam2go, Gene Ontology classifications was assigned to 20,597 transcripts. Annot8r was used to provide KEGG orthology to our transcript dataset. A total of 25,292 KEGG pathway assignments were defined and further confirmed with reciprocal blast against the NCBI nr protein database. This has identified many *P. hawaiensis* gene orthologs of key conserved signalling pathways involved in development. We also generated small RNA sequences from *P. hawaiensis*, identifying 55 conserved miRNAs. Sequenced small RNAs that were not annotated by stringent comparison to mirBase were used to search the *Daphnia pulex* for possible novel miRNAs. Using a conservative approach, we have identified 51 possible miRNA candidates conserved in the *Daphnia pulex* genome, which could be potential crustacean/arthropod specific miRNAs. Our study presents gene and miRNA discovery in a new model organism that does not have a sequenced genome. The data provided by our work will be valuable for the *P. hawaiensis* community as well as the wider evolutionary developmental biology community.

## Introduction

Comparative functional studies to establish the evolution of developmental processes underpinning morphological diversity in animals have been limited to a few taxa in a few branches of the animal phylogenetic tree. One of the main limitations to further functional comparisons is the paucity of animal model systems amenable to detailed functional study. *P. hawaiensis*, an amphipod crustacean that is a member of the class malocrustraca [1], [2], [3], is one such model system waiting for more widespread exploitation [4].

*P. hawaiensis* is easily cultured and the whole life history is open to experimental study, a particular highlight is the amenability of the embryos to imaging and experimental manipulation. Due to the concerted efforts of key groups, we now have detailed knowledge of the *P. hawaiensis* embryology [5], an expanding set of molecular tools for transgenesis [6], [7], as well as methods for RNAi [8] and exquisite spatial expression analysis [9]. These will facilitate functional genetic studies in *P. hawaiensis* with the potential to make key contributions to our understanding of developmental evolution. Furthermore, the *P. hawaiensis* embryo presents a useful model system in itself to study the molecular mechanisms for early cell fate determination [10], [11]. Cell lineage is defined as early as eight cell embryos following holoblastic and asymmetrical cleavages and so far little is known about the genetic control of asymmetry and lineage mechanisms. For these reasons *P. hawaiensis* would be expected to rank higher as a model organism of choice for both developmental and comparative developmental studies.

Studies in new model organisms suffer largely due to lack of molecular techniques but also through lack of genomic and gene information. The latter remains a problem for *P. hawaiensis* with a paucity of genetic information in publicly available databases. The development of next generation sequencing technologies has enabled a rapid and cost effective generation of gene sequence in the form of ESTs and small RNA sequence data, which can provide us information on the transcriptional repertoire of any given life stage of an organism. Roche 454 pyrosequencing has the ability to generate sequence with long read lengths of typically 350–500 bp enabling efficient and accurate *de novo* assembly [12], [13]. Alternatively, massively parallel short tag sequencing offers an even cheaper per base pair alternative [12], [13].

Here, we describe the sequencing and annotation of the early *P. hawaiensis* embryo transcriptome using Roche 454 GS FLX titanium next generation sequencing platform. We describe the assembly and annotation of the sequenced transcripts using Pfam, GO and KEGG databases. We have carried out comparative analysis to evaluate the extent of gene conservation between *P. hawaiensis* and 17 other species. Furthermore we have characterised and presented the *P. hawaiensis* homologs of genes involved in developmental signalling pathways that are present in our dataset. Using short read sequencing we have also sequenced the small RNA fraction of the developing *P. hawaiensis* embryo at two different stages to identify and analyse the expression of conserved mature miRNAs. We have also attempted to characterise miRNAs conserved between *P. hawaiensis* and *Daphnia pulex* by homology mapping sequenced small RNAs to the Daphnia genome. Together our data provide an important resource for the *P. hawaiensis* research community and those studying developmental evolution in the Arthropoda.

## Results and Discussion

### Pyrosequencing and Transcriptome assembly

**Figure 1:**
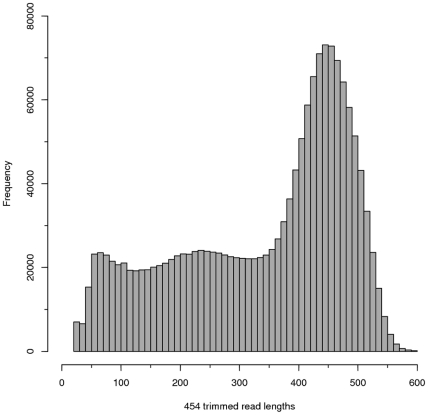
454 read length distribution. A plot showing the distribution of the trimmed *P. hawaiensis* cDNA sequence reads generated from 454 Titanium sequencing chemistry.

We set out to define the transcriptome of *P. hawaiensis* during early development and increase the current number of known genes in this species. We extracted total RNA from embryos up to day 3.5 in age (Germ band stage) [5]. Extracted *P. hawaiensis* total RNA was depleted for Ribosomal RNA and dscDNA library was constructed using smarter cDNA synthesis kit. Libraries were normalised using double stranded DNA specific nuclease (DSN) and sequenced using a Roche 454 GS Titanium sequencer. Pryosequencing of *P. hawaiensis* cDNA library yielded a total of 1,733,121 reads with a mean length = 336 bp (Figure 1), of these 1,618,531 passed the requirements for assembly by Newbler v2.5. A total of 1,320,592 (76.2%) were successfully assembled into 62,267 isotigs and contigs belonging to 41,013 isogroups. The average Isotig length was 1,099 bp (N50 = 1,293 bp).

Reads not incorporated into the assembly, referred to as singletons, have been shown to contain potentially useful transcript information [14], [15]. Accordingly, these singleton reads were processed further into subsets of lower confidence transcripts for inclusion in the analysis. The 220,317 unassembled singletons were assembled by CAP3 into 34,291 contigs (≥200 bp), with 63,987 singlets (≥200 bp) remaining. The average CAP3 contig and singlet lengths were 446 bp and 373 bp respectively.

This resulted in a combined dataset comprising 160,545 transcripts with an average length of 670 bp (N50 = 823 bp).

**Figure 2:**
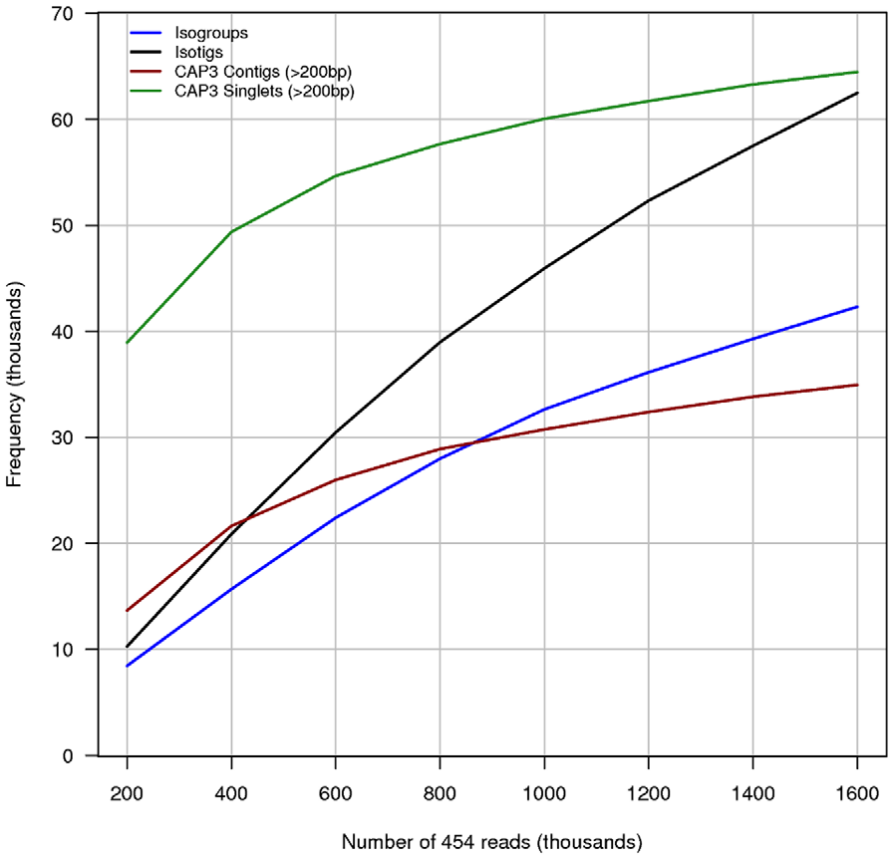
Transcriptome assembly metrics according to the number of input reads. The complete transcriptome assembly method was applied to an increasing number of randomly selected reads from the 454 sequencing data. With increasing reads the change in Isogroup (gene) discovery decreases in relation to that of Isotigs (transcript isoforms). This suggests further sequencing would define more alternately spliced transcripts in relation to fewer additional genes. The relative proportion of singlet and CAP3 contig transcripts also decreases as increasing read coverage of the transcriptome allows these low coverage transcripts to be assembled into isotigs.

We analyzed the number of transcripts assembled by Newbler and then by CAP3 against an increasing number of reads selected randomly from the total read set (Figure 2). As more reads are included the proportion of CAP3 singlets and contigs decreases compared to the proportion transcripts defined by Newbler. This represents a decrease in the proportion of unique reads (no similarity to any other read) as sequencing depth increases. Furthermore, the proportion of Newbler Isotigs increases at a greater rate than for Isogroups (theoretical genes) indicating that more transcript variants are defined than novel genes with increasing numbers of reads from the embryo stage library. This suggests that even deeper sequencing with this 454 library might still yield additional novel transcripts, but that sequencing from other *P. hawaiensis* life stages would be a more appropriate strategy for the identification of more *P. hawaiensis* genes.

### Coding content assessment through open reading frame (ORF) analysis of Isogroups

**Figure 3:**
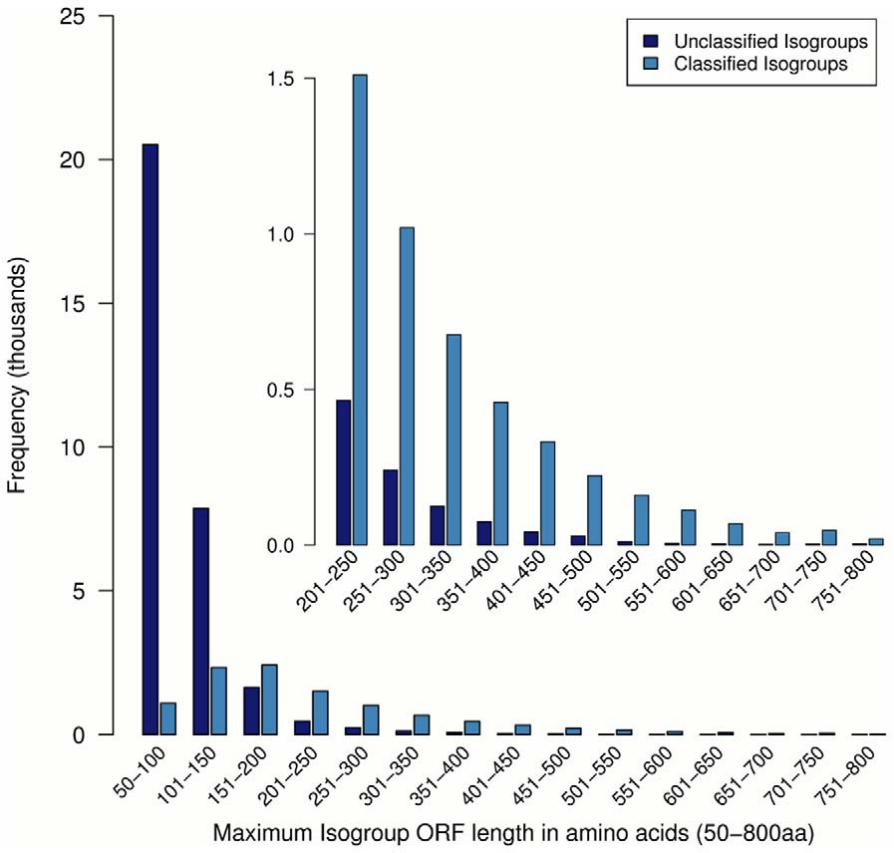
Isogroup maximum open reading frame length distribution. The distribution of the longest putative protein coding region in each Isogroup. Open reading frames are defined as translated regions that are free of stop codons. The frequency of ORFs according to length and Isogroup homology classification are shown. A classified Isogroup confers a homology match to Pfam, a selected proteome, or to the KEGG and GO databases via Annot8r. Isogroups with a maximal ORF length less than 150aa are typically shown to have no determined homology to known proteins, while those with longer ORFs, as shown in the subplot, are more frequently classified.

We assessed the coding content of the transcript set by performing a simple ORF analysis on assembled Newbler isogroups. We found the longest ORF in each isotig defined by both start and stop codons, or by just by stop codons alone (Figure 3). We also classified the ORFs by whether they were classified (C) by various annotation procedures or if they remained unclassified (U). We describe our annotation procedures and analysis in subsequent sections).

The large number of isogroups with an ORF ≤100 amino acids in length, particularly for the group containing both start and stop codons suggests that many transcripts may not represent coding regions of the *P. hawaiensis* transcriptome. Nevertheless, the number of all isogroups with ORFs >150 amino acids that have been given a classification by our annotation approach suggests that the majority of these represent protein coding genes.

### Classification and comparative proteome analysis of the assembled *P. hawaiensis* transcriptome

**Figure 4:**
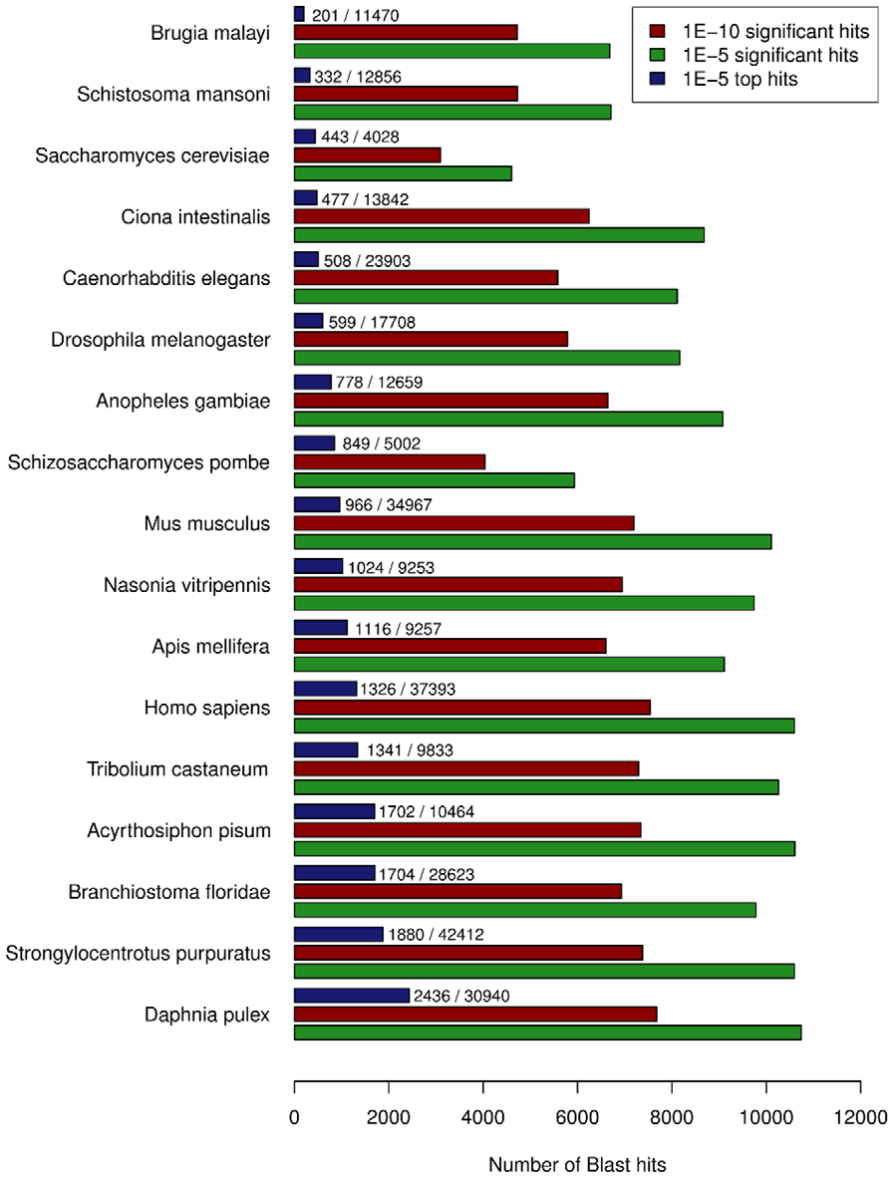
Cross-species BLASTX results. The cross-species BLASTX results for *P. hawaiensis* transcripts compared to selected proteomes. The frequencies of transcript matches to each species with a significant hit (E-value ≤1e-5 green bar, ≤1e-10 red bar) are shown. The frequencies of top transcript matches (blue bar) compared to the total number of proteins are also shown.

Classification of our assembled transcriptome was carried out using four different analyses to define the total number of transcripts with homology. Firstly, a comparison of the sequenced *P. hawaiensis* transcripts was carried out with proteomes from a selection of 17 different species. The homology of the transcripts to each proteome was assessed using BLASTX with significance E-value thresholds of 1e-5 and 1e-10 (Figure 4) (The blast results for cross-species homology matches are provided as Table S1). The total number of transcripts with significant matches was 17,683 and 12,271 for each of the respective thresholds. Of the species compared, the proteome of the crustacean *Daphnia pulex* showed the closest overall homology with 2,436 transcripts most closely matching *D. pulex* proteins with an E-value of ≤1e-5.

Secondly, transcripts were analysed for homology to Pfam protein families. Six-frame translations of all transcript sequences were scanned for matches to Pfam protein domains by applying the HMMER 3.0 algorithm against the Hidden Markov Models (HMM) of PfamA version 24 [16]. Each significant match (E-value ≤1e-5) was recorded. This provided 27,815 Pfam classifications for 22,648 (14.11%) of all transcripts.

**Figure 5:**
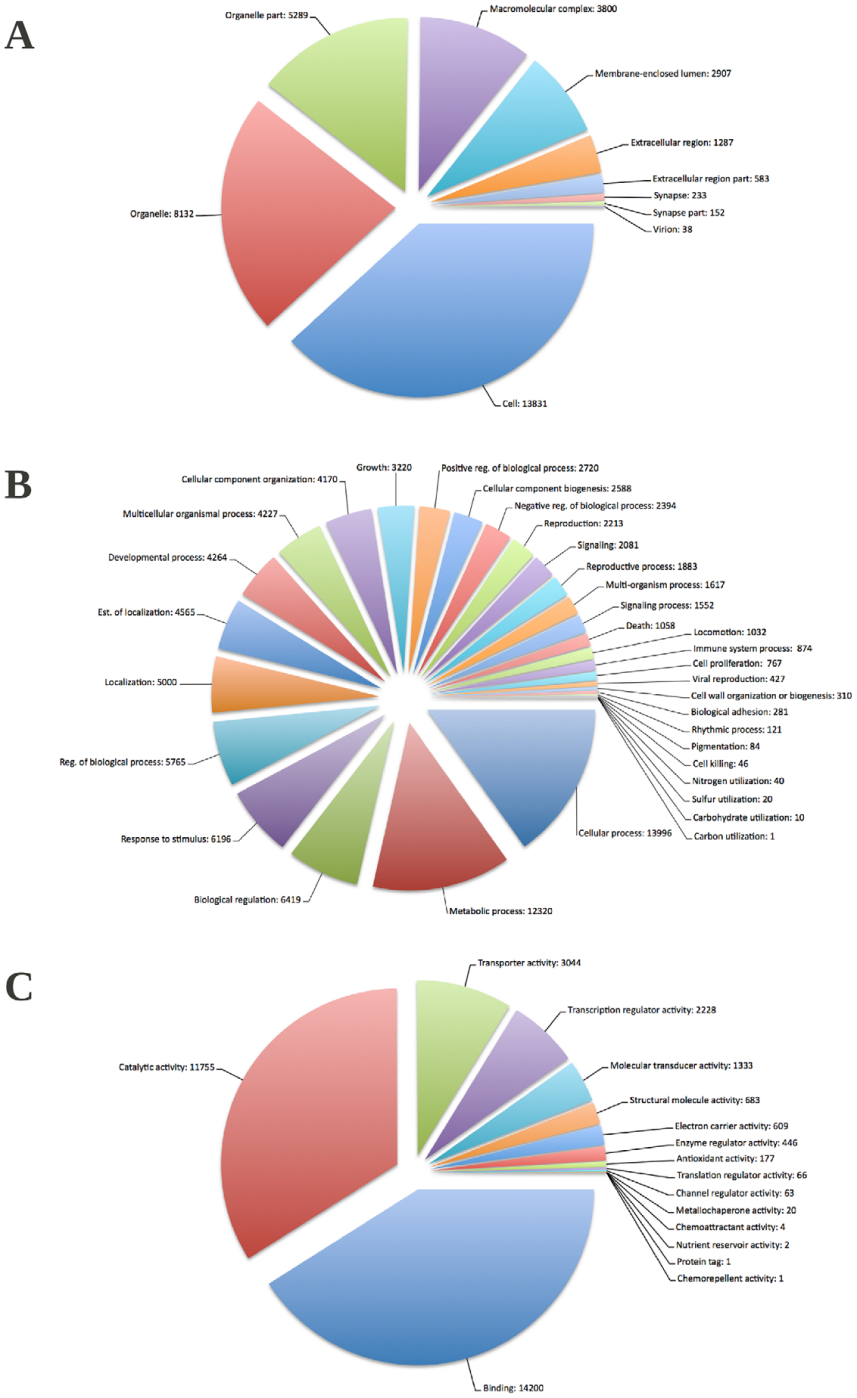
The classifications of transcripts according to Gene Ontology (GO) terms. The classification counts are shown for each of the first tier terms of the three GO database domains; Cellular Component (A), Biological Process (B), and Molecular Function (C).

A third analysis utilised the Annot8r classification pipeline [17] where transcripts were classified against the Gene Ontology (GO) database [18] comprising of non-redundant invertebrate protein sequences from NCBI and additional proteomes of species used in the cross-species BLASTX analysis. Annot8r GO classification resulted in 12,936 (8%) of the total transcripts assigned with 280,978 GO terms. The GO database represents ontological descriptions for gene products according to three main groupings: associated biological processes, cellular components and molecular functions. The hierarchical structure of GO terms allowed transcript classification to be summarised further to first tier terms of the three domains of the GO database (Figure 5A, B, and C).

By implementing the predefined association table of GO codes and Pfam domains, pfam2go (http://www.geneontology.org/external2go/pfam2go), additional GO term assignments could be made to Pfam matched transcripts. This provided assignments to 16,212 (79%) of the Pfam classified transcripts. Combined, Annot8r and pfam2go conferred GO terms to 20,597 (13%) of all the transcripts; 15% of Isotigs, 16% CAP3 contigs, and 9% of singlets.

**Figure 6:**
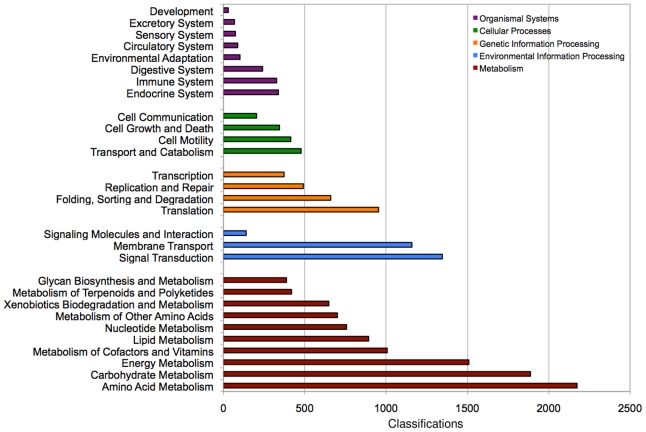
The classification frequencies of transcripts according to Kyoto Encyclopaedia of Genes and Genomes (KEGG) pathways. Classifications were determined by Annot8r for all transcripts. Shown are the classification counts for each of the first tier pathways under the 5 main KEGG categories.

We were interested in identifying homologs of conserved developmental genes in our transcriptome as these are likely to be largest initial interest to the community. Therefore, using the Annot8r pipeline we were able to provide KEGG orthology and pathway classifications [19] for 9,964 (6%) of the transcripts; 7% of Isotigs, 8% CAP3 contigs, and 4% of singlets (Figure 6). In total, 25,292 KEGG pathway assignments were defined. These annotated sequences were then used in a reciprocal blast analysis against the NCBI NR protein sequence collection to establish orthology. This analysis identified a number of genes with developmental roles in *P. hawaiensis* and other animals (see Table S2 for selected examples).

In our dataset only 29,668 of all transcripts (18.5%) were classified where orthology according to the classification methods used here. However, this is comparable to other invertebrate transcriptomes sequenced with 454 technology [14], [15], [20], [21], [22]. One possible explanation for the small proportion of classified transcripts with orthology in our dataset could be a result of our dataset consisting of large amounts of 3′ UTR and/or sequence not containing conserved domains. Our method of library construction used oligo dT priming, will have a 3′ bias, as opposed to random priming. In addition *P. hawaiensis* is known to have a large genome (∼3.6 Mb) with large intergenic and intronic regions [23]. It is possible that many transcripts may also have large UTRs by analogy with large intergenic and intronic regions.

### Direct comparative analysis against the *Daphnia pulex* genome

Given that 35,317 transcripts (including CAP3 contigs and singlets) did not provide a significant BLASTX match to the animal proteomes examined, but nonetheless encode an ORF of 100aa or more, and that of the 17,683 transcripts that did provide a proteome match (Figure 4), some 10,740 where to the other crustacean *D. pulex*, we considered the possibility that there might be a significant number of novel or rapidly evolving genes in our dataset.

To test this hypothesis we queried the genome sequence of *D. pulex* directly, as this might help define shared pan-crustacean sequences that remain absence from the proteomes we used.

First, we identified 237 transcripts encoding an ORF ≥100 amino acids in length that did not align to animal proteome sequences, but did align to the genome sequence of *D. pulex* through TBLASTX (E-value threshold: 1e-10). Of these, 209 transcripts had annotation through Pfam or Annot8r, while 28 had no classification. Of the annotated transcripts we found 76 that matched the *D. pulex* at a higher E-value threshold of 1e-20, strongly suggesting that these transcripts are protein-coding genes that are present in both crustaceans. These transcripts include genes with Pfam domains such as multi-antimicrobial exclusion protein (MATE) and member of the AcrB/AcrD/AcrF integral membrane protein family (Table S3) and demonstrate that many *P. hawaiensis* transcripts may encode genes that are not broadly phylogenetically conserved or indeed potentially novel. Additionally, of the 28 unannotated transcripts, 12 had a very high identity with regions of the Daphnia pulex genome (E-value: ≤1e-20). Taken together these data suggest that our transcriptome contains protein coding genes that may be conserved in the pan-crustacea but not more broadly. Further analyses will make use of the *P. hawaiensis* data and other datasets to inform the identification of novel crustacean genes.

### *P. hawaiensis* micro RNA sequencing and annotation of conserved miRNA

Two small RNA libraries representing different stages of *P. hawaiensis* development were constructed. Firstly, a small RNA library from total RNA extracted from embryos collected at stage 1 (1cell; 0–4 hrs) to stage 4 (8cell; 7.5–9 hrs) was constructed (“early” stage). Secondly, a small RNA library from embryos post stage 4 to stage 14 (Germ band stage, gastrulation; 80 hrs) was also constructed (“late” stage). Solid sequencing resulted in 63,862,118 and 64,860,146 reads for each stage respectively. After trimming 10,111,821 (average length = 23.3 bp) and 11,519,655 (average read length = 23.6 bp) reads were used for the early and late stages respectively. Reference mapping to known mature miRNA sequences from mirBase (release 17) and tag counts analysis was conducted using the CLC small RNA analysis pipeline. Collectively, we identified 55 conserved *P. hawaiensis* miRNA sequences from the early and late stages of the *P. hawaiensis* embryogenesis (Tables S4 and S5). Only the identified miRNAs with tag counts greater than 100 in at least one of the two libraries have been listed in Table 1.

**Table 1:** Conserved miRNAs with high expression values identified in *P. hawaiensis* developing embryos.

Sequence	Early stage sequenced tag counts	Late stage sequenced tag counts	mirBase miRNA	mirBase reference species
AATTGCACTCGTCCCGGCCT	19011	2867	mir-92	*Daphnia pulex*
TCAGGTACTTAGTGACTCT	1362	480	mir-306	*Drosophila melanogaster*
AATGGCACTGGAAGAATTCACGG	877	1449	mir-263	*Anopheles gambiae*
ATGACTAGATCCACACTtATCCA	324	2635	mir-279	*Daphnia pulex*
TATCACAGCCACCTTTGATGAGCT	202	724	mir-2a	*Ixodes scapularis*
GTGAGCAAAGTTTtAGGTGTG	106	129	mir-87a	*Tribolium castaneum*
TGAAAGACATGGGTAGTGAGACG	90	197	mir-71	*Bombyx mori*
TACTGGCCTGCTAAGTCCCAA	12	528	mir-193	*Daphnia pulex*
CAATGCCCTTGGAAATCCC	2	223	mir-2788	*Bombyx mori*
CTAAGTACTGGTGCCGC**a**	0	305	mir-252	*Lottia gigantea*

The table lists 10 small RNA sequenced from *P. hawaiensis* embryos with identified homology to known miRNA sequences present in mirBase (release 17). Bases in bold and lower case indicate the allowed mismatch during homology mapping. Tag counts refer to the number of sequence matches found to that particular reference miRNA sequence. Only the sequences greater than 100 tag counts for either of the two stages examined has been listed. All identified miRNAs are presented in Tables S4 and S5.

We identified both mir-10 and mir-100 in *P. hawaiensis* that are conserved across the Eumetazoa. Among the other conserved miRNA identified in *P. hawaiensis*, we have found 9 miRNA families among the 30 known conserved miRNA across Bilateria and throughout animal evolution. These include mir-1, mir-9, mir-34, mir-79, mir-92, mir-184, mir-193, mir-263a and mir-317 [24], [25]. We were able to look at the abundance of particular miRNAs during the two developmental stages, which are reflected by the number of sequenced reads representing the miRNA (tag counts; [26]). Members of the miR-92 family were found to be most abundantly expressed in both the *P. hawaiensis* stages sequenced. The exact function of miR-92 is not known in protostomes, but in both developing drosophila [25] and Tribolium embryos [27], members of mir-92 family were also found to be expressed at high levels. Using *in situ* hybridisation against nascent transcripts, the expression of miR-92a was detected in the brain primordia and a subset of ventral never cord in Drosophila germband stage embryos [28], which would infer a function for miR-92a in the development of the drosophila central nervous system. In contrast, miR-92 is associated with cancer progression in humans, and it is encoded in the larger miRNA 17–92 cluster [29]. mir-92 and family members were shown to be involved in cell proliferation and found to be overexpressed in several cancers such as medullobastoma [30], breast cancer, where it down regulates estrogen receptor β1 (ERβ1), which is known to have tumour suppressor properties [31] and hepatocellular carcinoma [32]. Our data suggest that mir-92 may have a role in early *P. hawaiensis* embryogenesis.

Protostomes are known to have 12 conserved miRNA families [24], [33] we were able to identify 10 (bantam, mir-2, mir-12, mir-87, mir-275, mir-276, mir-279 and mir-307, mir-750, mir-1175) in *P. hawaiensis*. The two families (mir-76 or mir-981 and mir-1993) that were not identified may be expressed during later developmental stages. We were also able to identify mir-iab-4 which is specific for arthropod lineage and regulates the expression of *Ultrabithorax* during drosophila development. In the early *P. hawaiensis* embryos, miR-2 was found to be abundantly expressed. In Drosophila, there are eight almost identical members of the mir-2/mir-13 family clustered in 4 distinct genomic loci. Each cluster has a spatially differing expression pattern in the developing drosophila embryo [28]. Based on evidence of translational repression of the target genes hid, grim, skl and rpr, mir-2 and mir-13 are thought to be involved in the regulation of apoptosis [34], [35]. In *Bombxy mori*, mir-2/mir-13 family members are encoded in two clusters. In addition a new member of the family, mir-2b was identified encoded in one of the clusters consisting of mir 2a-1/2a-1*/2a-2/2b/13a/13b [36]. However, we could not detect mir-13 family members in our sequencing results, which could mean either the mir-13 sequence is not conserved or present in *P. hawaiensis* but it could also mean that mir-2 and mir-13 are not clustered together in *P. hawaiensis*. There is evidence elsewhere of miRNA loss, for example in *Anopheles gambiae* only 5 members of this gene family are present in just a single cluster [37].

Another example of loss of miRNA family is mir-71, which is known to be lost from *Drosophila* [38]. We identified *P. hawaiensis* mir-71, which is known to have homologs in other invertebrates including *Tribolium* [27].

*P. hawaiensis* mir-306 and mir-92 were found to be highly expressed in the 8-cell embryos (Table 1). Importantly the initiation of zygotic expression in *P. hawaiensis* embryo is sometime after the 8-cell embryo and before the 16-cell embryo stage [39], therefore the mature miRNAs identified in the early embryos (8 cell or earlier) must have been maternally provided. Previously it was found that in *Drosophila* mir-306 is also maternally deposited [35], [37]. The miRNAs identified in early *P. hawaiensis* embryos could be highly important in shaping the translational landscape of the early embryo and thus may play a vital role in early cell fate determination.

### Crustacean specific miRNAs: homology mapping to the *Daphnia pulex* genome

Among the identified conserved miRNAs in *P. hawaiensis* mir-996, which is conserved in the pancrustacea lineage was also present. Furthermore, among the annotated *P. hawaiensis* miRNAs we identified 22 out of the 45 previously characterised miRNAs in *D. pulex* [Table 2; [33]. Subsequently, to identify novel crustacean specific miRNAs, we used the MapMi annotation pipeline [40] to search the recently published *D. pulex* genome [41] for homology matches to the sequenced small RNA from *P. hawaiensis.* For this analysis, we used sequenced small RNAs from *P. hawaiensis* that were not annotated by CLC and showed no sequence homology to conserved miRNAs in mirBase. The unannotated small RNA sequences were filtered for known *P. hawaiensis* rRNAs and tRNAs, in addition the unannotated small RNA sequences were also filtered for all *D. pulex* rRNA, tRNA and ncRNA sequences. As positive controls we used the 22 conserved miRNA sequences to map back against the *D. pulex* genome using the MapMi default settings, which allows for 1bp mismatch. All 22 conserved miRNAs were identified in the *D. pulex* genome at the correct chromosomal locations as indicated in mirBase. We identified 51 *P. hawaiensis* small RNA sequences that mapped through sequence homology to the *D. pulex* genome with a conservative MapMi score threshold of ≥40. In order to ensure the MapMi identified sequences did not contain sequence homology to any other known miRNA sequences in mirBase further analysis was performed. We performed BLASTN with the 51 novel sequences identified by MapMi against the known miRNA sequences in mirBase. For short sequence blast analysis BLASTN was executed with the PAM30 matrix and with no significance threshold. The BLASTN analysis identified 13 unique matches to mirBase, which were further visually inspected using ClustalW2 multiple sequence alignment tool to examine sequence identity to miRNA seed sequences [24]. Out of the 51 identified sequences, 2 matched the seed sequences of known miRNA with 1 bp mismatch and given that many miRNAs share seed sites may represent miRNAs in the same family. These were Ph_novel_34 and Ph_novel_44 which matched to *Capitella teleta* miR-2713 (Accession number, MIMAT0013582) and *Bos taurus* miR-2462 (Accession number, MIMAT0012049) respectively. The 20 unique small RNA sequences from *P. hawaiensis* that mapped to the *D. pulex* genome with the highest MapMi scores are given in Table 3 (Complete list with expression values are listed in (Table S6).

**Table 2:** MapMi homology mapping of *P. hawaiensis* conserved miRNA to the *Daphnia pulex* genome.

mirBasemiRNA	Query sequence	Mismatch	Chromosome	start	end	length	MapMi Score
mir-1	TGGAATGTAAAGAAGTATGGAGC	0	scaffold_1	1720882	1720904	23	48.27
mir-2a	TATCACAGCCAGCTTTGATGAGC	0	scaffold_80	241083	241105	23	54.12
mir-12	TGAGTATTACATCAGGTACTGGT	0	scaffold_1	1847883	1847905	23	47.86
mir-34	TGGCAGTGTGGTTAGCTGGT	0	scaffold_4	1242095	1242114	20	52.09
mir-71	TGAAAGACATGGGTAGTGAGATG	0	scaffold_80	240430	240452	23	57.26
mir-79	ATAAAGCTAGGTTACCAAAG	0	scaffold_2	1526251	1526270	20	51.97
mir-92	AATTGCACTCGTCCCGGCCTGT	1	scaffold_38	876194	876215	22	35.05
mir-193	ACTGGCCTGCTAAGTCCCAA	0	scaffold_167	85458	85477	20	46.75
mir-252a	CTAAGTACTGGTGCCGCAGGAG	1	scaffold_285	66066	66087	22	37.02
mir-263b	AATGGCACTGGAAGAATTCAC	0	scaffold_87	475620	475640	21	31.44
mir-263a	CTTGGCACTGGAAGAATTCACA	0	scaffold_87	475817	475838	22	45.22
mir-276	AGGAACTTCATACCGTGCTCTC	0	scaffold_15	755668	755689	22	42.58
mir-279	ATGACTAGATCCACACTC	0	Scaffold_63	523658	523675	18	35.68
mir-307	TCACAACCTCCTTGAGTG	0	Scaffold_47	601105	641122	18	33.14
mir-317	TGAACACAGCTGGTGGTATCTCA	0	scaffold_4	1243963	1243985	23	52.54
mir-745	GAGCTGCCCAGTGAAGGGCA	1	scaffold_63	361999	362018	20	34.70
mir-965	TAAGCGTATGGCTTTTCCCCT	0	scaffold_32	27783	27803	21	48.07
mir-993	GAAGCTCGTTTCTACAGGTATCT	0	scaffold_7	282360	282382	23	49.27
mir-iab-4-5p	ACGTATACTGAATGTATCCTGA	0	scaffold_7	515547	515568	22	44.98
mir-iab-4-3p	CGGTATACCTTCAGTATACGTA	0	scaffold_7	515582	515603	22	42.92
bantam	GAGATCATTGTGAAAGCTGAT	0	scaffold_115	370210	370230	21	44.47
mir-275	TCAGGTACCTGATGTAGCG	1	scaffold_4	1790782	1790800	19	44.53

Table shows the result of MapMi homology mapping to the *D. pulex* genome with *P. hawaiensis* sequences annotated by known miRNA from mirBase with a tolerance of a 1 bp mismatch. The mapping position on the *D. pulex* chromosome corresponding to known miRNA sequences are shown with the calculated MapMi score for the mapping and RNA fold analysis.

**Table 3:** MapMi homology mapping of *P. hawaiensis* novel small RNA sequences to the *Daphnia pulex* genome.

Sequence name	Sequence	Mismatch	Chromosome	Start	End	length	MapMi Score
Ph_novel_1	AGGTTAGGTAATGTTAGGTTAG	1	scaffold_5153	33927	33948	22	62.10
Ph_novel_2	TGCGAGAAGTACGGGGATC	0	scaffold_4250	5278	5296	19	53.24
Ph_novel_3	GGCGTTCTTGGCTGCGTTA	1	scaffold_11	2121025	2121043	19	53.06
Ph_novel_4	CTTGAGTCCTGAGTGGACG	1	scaffold_64	451499	451517	19	50.77
Ph_novel_5	TTGAGTATGTCCTCGCGAGT	1	scaffold_49	256891	256910	20	50.01
Ph_novel_6	AGGAGCCGTTGTCGTCCG	0	scaffold_1	1336698	1336715	18	48.91
Ph_novel_7	TCTTTGGTGGTTTAGCTGTA	1	scaffold_32	69614	69633	20	48.81
Ph_novel_8	TTGGGGTTGCTTTAGTGAG	0	scaffold_13	788021	788039	19	48.52
Ph_novel_9	GGCCGCGTGATGAGCGCG	1	scaffold_25	860431	860448	18	47.38
Ph_novel_10	TTGAAAACCTGCAGCTGTCCCGT	1	scaffold_74	237849	237871	23	46.50
Ph_novel_11	CCGTTGCGCTGTCGCGCC	1	scaffold_49	84010	84027	18	46.30
Ph_novel_12	TCCATCGACGGAAGATCTC	1	scaffold_19	972093	972111	19	46.23
Ph_novel_13	GGGGCGGTACATCTGTCAAACGA	0	scaffold_1482	7141	7163	23	46.06
Ph_novel_14	TGCATTCGTTCAGGCTGCA	1	scaffold_27	880161	880179	19	45.80
Ph_novel_15	GAGAGTCTGCCCGCCTTG	1	scaffold_8	428158	428175	18	45.20
Ph_novel_16	CCGGAAGGCCGGCACACTGG	1	scaffold_11159	299	318	20	45.05
Ph_novel_17	GTTCCAGGCTACGCTCGTC	1	scaffold_2	2005051	2005069	19	44.21
Ph_novel_18	CGGGGCTTCCTCGACTAGA	1	scaffold_17	706560	706578	19	44.04
Ph_novel_19	TAGGCGTGCCGATGCAAG	1	scaffold_123	175996	176013	18	43.96
Ph_novel_20	CTACTTACTGCTGGCGGT	1	Scaffold_14	941690	941707	18	43.90

20 *P. hawaiensis* small RNA sequences identified by MapMi as potentially novel miRNA sequences conserved with *D. pulex*. Sequences mapped to the *D. pulex* genome but shared no homology to known miRNAs. A conservative MapMi score threshold of ≥40 and a mismatch tolerance of 1 bp were used. The mapping coordinates to the *D. pulex* chromosome are presented. All 51 potential novel miRNA sequences and their expression values are detailed in Table S6.

We analysed the genomic position of the potentially novel miRNAs conserved between both crustaceans to see if these were clustered with each other or with conserved *D. pulex* miRNAs. Only two examples were found where potential novel miRNA sequences were proximally located such that they are likely to be miRNA clusters; Ph_novel_7 was found 968 bp downstream on the same chromosomal scaffold as dpu-mir-279b and Ph_novel_25 and Ph_novel_42 were found 1,739 bp apart on scaffold_461. Finally, Ph_novel_40 and Ph_novel_48 were found to be only 14 bp apart thus this is evidence that these two sequences could be miRNA and miRNA* in the same hairpin. Overall our data suggest that we have identified the sequences of many widely conserved miRNAs, as well as miRNAs conserved between two crustacean species.

### Conclusion

We have utilised the long read length sequence output by Roche 454 pyrosequencing to generate, assemble and annotate the trascriptome during the early developmental stages of the amphipod crustacean *P. hawaiensis*. Recently, Zeng *et al*. [42] also published a *P. hawaiensis* 454 transcriptome from ovary and embryo developmental stages assembled from more than 3 million reads. While a similar number of transcripts were classified through homology searches in each study, Zeng et al. have identified a fewer number of Isotigs (62,267) and Isogroups (35,301). The combination of both datasets would likely result in a transcriptome assembly superior to both studies.

We have also used the SOLiD analyser 4 to generate sequences of the small non-coding RNA fraction during early development stages. Small RNA sequence generated was homology mapped to identify conserved miRNAs and identify potential novel miRNAs that may be specific to the crustacean/arthropod lineage. As an emerging model-organism for comparative developmental studies, the sequence information we have generated should enhance further functional studies in *P. hawaiensis*.

The assembled trancriptome in FASTA format, accompanied by all the annotation results generated in this study, are available through anonymous FTP: ftp://ftp.nottingham.ac.uk/pub/Projects/deepseq/Parhyale.

## Materials and Methods

### *P. hawaiensis* culture

*P. hawaiensis* cultures were maintained in the laboratory in 20 L plastic boxes in approximately 2 L of artificial sea water (33 g/L Tropic Marine sea salt in deionised water) and crushed corals. The cultures were aerated and housed in incubators at 25°C. The cultures were fed with crushed fish flakes once every week and cleaned every week.

### Embryo collection, staging and Total RNA extraction

*P. hawaiensis* pairs were isolated and cultured separately in small plastic containers. Separated fertilized females carrying embryos were anesthetised with Clove oil (1∶10000 diluted with artificial sea water). Embryos were teased out from the female ventral brood pouch with soft tweezers and blunt-ended needle under a light microscope. Embryos were staged according to development and only embryos that had developed to stage 13 (72 hrs) and younger were collected (Browne *et. al.* 2005). Approximately 800 embryos were collected and frozen in Trizol (Life Technologies, Cat No. 155966) at −80°C. Embryos frozen in Trizol were homogenized with a pestle and Trizol was added to a volume of 1 ml then total RNA was extracted as described by the manufacturer. Total RNA was resuspended in 19 µl of 1× turbo DNase buffer (Ambion, AM2238). 2 U of turbo DNase was added and incubated at 37°C for 30 minutes. Turbo DNAse was inactivated by phenol, pH 4.3: chloroform (50∶50; Sigma, Cat No. P4682-100 ML). Total RNA was alcohol precipitated overnight at −80°C. Precipitated total RNA was centrifuged at 12,000× g for 15 minutes at 4°C, washed with 70% ethanol, air dried and re-suspended in 10 µl of nuclease-free water.

### 454 whole transcriptome library Preparation and sequencing

Approximately 10 µg of total RNA from *P. hawaiensis* embryos was used for the enrichment of mRNA with Eukaryote Ribominus kit (Invitrogen, Cat No. A10837-08). 200 ng of ribominus RNA was used to make double stranded cDNA using the SMARTer PCR cDNA synthesis kit (Takara Biosciences, Cat No. 634925). Normalisation of the amplified transcriptome was carried out with Duplex Specific Nuclease (Evrogen, Cat No. NK001) as specified by the manufacturer. Amplified dscDNA (5 µg) was fragmented to an average size of 500 bp with Covaris S2 sonicator (fragmentation parameters: frequency sweeping mode, water bath temperature of 5°C, Duty cycle – 5%, Intensity – 3, cycles/burst – 200 and Time – 120 secs). Fragmented dscDNA was size fractionated on a 1.5% TAE gel and 400–900 bp fragments were excised and purified with QIAquick gel extraction columns (Qiagen, Cat. No 28760). Standard fragment library was constructed as described in the Roche general library preparation guide. Sequencing was performed on the Roche 454 GS FLX titanium sequencer according to the manufacturer's instructions.

The reads generated were submitted to the EBI Sequence Read Archive: http://www.ebi.ac.uk/ena/data/view/ERP001159.

### Solid Small RNA library preparation

Total RNA was extracted from staged embryos. Approximately 7 µg of total RNA was used. Small RNA enrichment was carried out using a flash-page fractionator (Ambion, Cat No. AM13100). Small RNA fragments <40 bp were isolated and cleaned using the flashPAGE Reaction Clean-up kit (Ambion, Cat. No. AM12200) as stated by the manufacturer. The size selected small RNA was used to prepare barcoded Solid small RNA libraries as stated in the Solid Total RNA Seq protocol (Applied Biosystems, Cat no. 4445374). Sequence was performed on Solid 4 analyser according to the manufacturer's instructions. The reads generated were submitted to the EBI Sequence Read Archive: http://www.ebi.ac.uk/ena/data/view/ERP001159.

### 454 Transcriptome assembly

Reads generated by pryosequencing were assembled using the Roche gsAssembler (Newbler) program version 2.5. Reads were initially trimmed against a sequence library of adaptor sequences used in the sequence library process as well as the removal of other sequencing artefacts. The remaining reads were then screened against previously defined rRNA sequences of *P. hawaiensis* and three *Daphnia* species, as well as a range of bacterial genomes that represented possible environmental contaminants. The remaining reads that met the minimal length requirement of 200 bp were then assembled by the Overlap-Layout-Consensus (OLC) methodology of Newbler.

Newbler initially assembles contigs from overlapping reads according to an estimated depth coverage calculation. The alignments of reads spanning these contigs are then used as to create transcripts, termed ‘isotigs’ by Newbler. Differential joining of the same group of contigs based upon read alignment evidence define different isotigs that are part of the same theoretical gene, or ‘Isogroup’. This is analogous to defining differently splice variants of the same gene sequence. An additional feature in this most recent version of the program allows the output of all reads that were accepted by Newbler in FASTA and QUAL format. In conjunction with the assembly read fate information, this allows for the simple identification of Newbler singleton reads for further processing.

The singleton reads in FASTQ format were input for the CAP3 [43] alignment algorithm that also uses an OLC methodology for aligning sequences. Transcripts (contigs) defined by CAP3 are dependant on overlap between two or more reads and not read coverage of the assembled transcript (contig). CAP3 contigs and CAP3 singlets (singleton reads not assembled by either Newbler or CAP3) were then screened by length at ≥200 bp. This was necessary in order remove the abundant short transcripts that frequently comprise low complexity sequencing artefacts that were not removed by the Newbler filtering steps as well as lacking the necessary base space information required for functional determination. The final transcript dataset comprises 3 subsets of transcript sequences; members of Newbler isogroups, CAP3 contigs, and unassembled read singlets.

### Transcript classification

The classification pipeline Annot8r [17] is able to assign gene function terms from the Gene Ontology (GO) database [18], and classifications for the molecular interaction pathway networks and gene orthology from the Kyoto Encyclopaedia of Genes and Genomes (KEGG) [19]. It is also able to assign Enzyme Commission (EC) codes. Transcript sequences were aligned by BLASTX to the GO, KEGG and EC manually curated entries of the Swiss-Prot sequence database (July 2011 release); Trembl sequences were excluded [44]. Processing of the BLAST results through the Annot8r PostgreSQL database allowed matching transcripts to inherit GO, KEGG and EC classifications. A significant alignment threshold of E-value 1e-10 was applied.

The complete six-frame amino acid translations of each transcript were tested for homology protein to Pfam-A protein families using the hmmscan algorithm applied to the Hidden Markov Model dataset (Pfam-A.hmm version 24) [16]. An E-value threshold of 1e-5 was applied to determine family homology of each transcript. Gene Ontology terms were assigned to Pfam matched transcripts via the pfam2go lookup table (http://www.geneontology.org/external2go/pfam2go). This procedure was implemented through a Perl script.

### Analysis of homology with other animals

The number of protein sequences defined for each species used in the cross-species homology analysis was as follows: *Daphnia pulex*: 30940, *Acyrthosiphon pisum*: 10464, *Strongylocentrotus purpuratus*: 42412, *Tribolium castaneum:* 9833, *Homo sapiens:* 37393, *Nasonia vitripennis*: 9253, *Mus musculus*: 34967, *Branchiostoma floridae*: 28623, *Anopheles gambiae*: 12659, *Apis mellifera*: 9257, *Ciona intestinalis*: 13842, *Drosophila melanogaster*: 17708, *Caenorhabditis elegans*: 23903, *Schistosoma mansoni*: 12856, *Brugia malayi*: 11470, *Schizosaccharomyces pombe*: 5002, *Saccharomyces cerevisiae*: 4028. All proteome sequences were sourced (March 2011) from the NCBI ftp server with the exception of *D. pulex* that was sourced from the DOE Joint Genome Institute. Transcript sequences were compared to a blast database of the proteomes using BLASTX. A minimum significant alignment threshold E-value of 1e-5 was used. BLAST results were processed using BioPerl scripts.

### Reciprocal Blast analysis

The sequences of transcripts selected based on their assigned KEGG classification of selected developmental pathways. These transcripts were then queried against the NCBI non-redundant (NR) protein dataset (March 2011) using BLASTX with a threshold E-value of 1e-10. Resulting blast results were manually selected for ortholog matches and tabulated.

### Analysis of small RNA sequence

Annotation of small RNA sequences were carried out with CLC genomics work bench using default settings. SAET adjusted solid reads were trimmed for adaptor sequences and the ‘tag counts’ programme was used to count number of sequence tags for sequences occurring > = 2. Reference mapping on clustered sequences was performed to annotate the small RNA sequences by mapping to the mature miRNA database using default settings allowing for 2 mismatches in colourspace (1 bp mismatch).

The MapMi [40] programme was used to detect potential homolog sequences in the Daphnia genome. The programme was used with maximum mature mismatch of 1 bp and a mismatch penalty of 10. Minimum score threshold MapMi score >40 was applied post analysis.

## Supporting Information

Table S1 **Blast results for cross-species homology matches.**
(XLSX)

Table S2 ***P. hawaiensis*** **gene orthologs for developmental signalling pathways.**
(DOCX)

Table S3 **Blast results of** ***P. hawaiensis*** **transcripts against the** ***D. pulex*** **genome.**
(XLSX)

Table S4 **Identifed conserved miRNAs and their expression values from early stage** ***P. hawaiensis*** **embryos (from 1-cell to 8-cell embryos).**
(XLSX)

Table S5 **Identifed conserved miRNAs and their expression values from late stage** ***P. hawaiensis*** **embryos (after 8-cell to germband stage).**
(XLSX)

Table S6 **MapMi homology mapping result of sequenced** ***P. hawaiensis*** **small RNA to the Daphnia pulex genome.**
(XLSX)
