# Supplementary material for: High Through-Put Sequencing of the Parhyale hawaiensis mRNAs and microRNAs to Aid Comparative Developmental Studies
Source: PLoS One. 2012 Mar 20;7(3):e33784. doi: 10.1371/journal.pone.0033784 (PMC3309017; doi:10.1371/journal.pone.0033784)
Supplement: Table S2 — P. hawaiensis gene orthologs for developmental signalling pathways. (DOCX) [file pone.0033784.s002.docx]

**Table S2: *P. hawaiensis* gene orthologs for developmental signalling pathways**

| **Orthologs** | **Number of hits** | | **Reciprocal blast result** |
| --- | --- | --- | --- |
|  | **Isogroups** | **CAP3 Contigs & singlets** |  |
| **Wnt** |  |  |  |
| Wingless /Wnt | 1 | 1 | WNT16 (EFX82994) [*Daphnia pulex*] 5e-53 |
| Armadillo/beta-catenine |  | 1 | Beta-catenin (ACH92925) [*Parhyale hawaiensis]* 2e-29  Armadillo (EFX85133) [*Daphnia pule*x] 4e-27 |
| Frizzled |  | 1 | Wnt-receptor frizzled-like protein variant 1 (EFX86267) [*Daphnia pulex*] 1e-29 |
| Tcf/Lef | 1 | 1 | Pangolin (XP 002572116) [*Schistosoma mansoni*] 3e-40  Transcription factor 7-like 1-A (NP 571344) [*Danio rerio*] 7e-38 |
| Axin2 | 1 |  | Regulator of G-protein signalling 3-like (XP 002741844) [*Saccoglossus kowalevskii* ] 9e-29 |
| **Notch** |  |  |  |
| Hairy and enhancer of split 1 | 1 | 1 | Enhancer of split mgamma protein putative (XP 002428313) [*Pediculus humanus*] 1e-44 |
| Anterior pharynx defective 1a homolog | 1 |  | Anterior pharynx defective 1a homolog isoform CRA_c (EDL85669) [*C. elegans*] 7e-14 |
| Corepressor interacting with RBJ1 | 1 |  | CBF1-interacting corepressor (EFN84618) [*Harpegnathos saltator*] 2e-67 |
| Deltex |  | 1 | Deltex 1 (NP 001081631) [*Xenopus laevis*] 8e-11 |
| Notchless |  | 2 | Notchless homolog 1 (Drosophila) isoform CRA_a (EAW80165) [*Homo sapiens*] 1e-65 |
| Strawberry notch | 1 |  | Protein strawberry notch [*Harpegnathos saltator*] 1e-57  Strawberry notch, isoform D [*Drosophila melanogaster*] 1e-52 |
| Nicastrin | 1 |  | mKIAA0253 protein (AK129102) [mus musculus] 4.9e-42 |
| **TGF-β** |  |  |  |
| Transacting transcription factor 1 (Sp1) | 2 |  | Sp1-4 protein (CBH30980) [*Parhyale hawaiensis*] 2e-33 |
| S-phase kinase associated protein 1 (skpA) | 1 |  | S-phase kinase-associated protein 1 (EFN63852) [*Camponotus floridanus*] 3e-71 |
| Mitogen activated protein kinase 1 | 2 |  | Mitogen-activated protein kinase ERK-A (XP 002428425) [*Pediculus humanus*] 1e-118  Mitogen-activated protein kinase kinase 1-like (XP 002733724) [Saccoglossus kowalevskii] 2e-94 |
| Decorin |  | 1 | Decorin, isoform CRA a (EAW97450) [*Homo sapiens*] 5e-47 |
| Activin-beta | 1 |  | Activin B (BAA83804) [*Anguilla japonica*] 6e-9  Activin beta B (CAA53636) [*Danio rerio*] 7e-9 |
| Roc1a/b | 2 |  | RING-box protein 2 (AC007973) [*Oncorhynchus mykis*] 2e-44  Ring box protein (NP 001040275) [*Bombyx mori*] 8e-57 |
| E1A binding protein p300 | 1 |  | E1A binding protein p300 (NP 808489) [*Mus musculus*] 4e-82 |
| SMAD specific E3 ubiquitin protein ligase 2 (Smurf2) |  | 1 | Ubiquitin protein ligase (XP 001658307) [Aedes aegypti] 3e-30 |
| Transcription factor Dp-1 |  | 1 | Transcription factor Dp-1 (EFN73759) [*Componotus floridanu*] 2e-31 |
| Short gastrulation protein (Sog)/ Chordin | 1 | 2 | Short gastrulation protein (ADO17754) [*Parhyale hawaiensis*] 3e-89 |
| Bone morphogenetic protein 8a (BMP8a) |  | 1 | BMP5-8 (ABC88372) [*Nematostella vectensis*] 4e-17  Bone morphogenetic protein 6 (AAH90689) [*Danio rerio*] 1e-16 |
| Lin-19-like (Lin19)/Cullin 1 |  | 1 | Cullin-3 (EFN72937) [*Camponotus floridanus*] 1e-52 |
| **Jak/Stat** |  |  |  |
| Akt1 |  | 1 | *Daphnia pulex* Akt1-like protein (EFX86288) 5e-16 |
| Growth hormone (Gh) | 1 |  | growth hormone-like protein 6 (AAU95543) [*Nomascus leucogenys*] 2e-88 |
| Phosphor-inositide-3-kinase (PI3-K21B) | 1 |  | Phosphatidylinositol 3-kinase regulatory subunit alpha (EFN63874) [*Camponotus floridanus*] 2e-31 |
| cyclin D2 (cycD2) | 1 | 1 | G1/S-specific cyclin-D2 (XP 002430897) [*Pediculus humanus*] 2e-14 |
| Cytokine inducible SH2 -contianing protein (Cish) | 1 |  | Cytokine-inducible SH2-containing protein (NP 989957) [*Gallus gallus*]2e-22 |
| Signal transducing adaptor molecule (Stam) | 2 |  | Signal transducing adaptor molecule (XP 002432444) [*Pediculus humanus*] 4e-37  signal transducing adaptor molecule 2 (NP 001007370) [*Danio rerio*] 2e-23 |
| E1A/CREB-binding protein (Nejire) | 1 | 1 | CREB-binding protein (XP 002423797) [*Pediculus humanus*] 5e-90  CREB-binding protein (EFN79936) [*Harpegnathos saltator*] 5e-28 |
| Son of sevenless (Sos) |  | 1 | Ras GTP exchange factor (XP 321917) [*Aedes aegypti* ras] 6e-58 |
| Protein tyrosine Phosphatase, Non-receptor type 6 (Ptpn6) |  | 2 | Protein tyrosine phosphatase, non-receptor type 2 (ABQ12941) [*Bos tauru*] 6e-26  Tyrosine-protein phosphatase non-receptor type 1 (EFN83906) [*Harpegnathos saltator*] 2e-26 |
| Suppressor of cytokine signalling at 36E (Socs36E) |  | 1 | Suppressor of cytokine signalling 5 (EFN79604) [*Harpegnathos saltator*] 3e-54  Suppressor of cytokine signalling at 36E, isoform A (NP 724096) [*Drosophila melanogaster*] 1e-40 |
| Thyroid peroxidase (Tpo) |  | 1 | Chorion peroxidase (EFN62091) [*Camponotus floridanu*] 4e-19 |
| **Nfκβ** |  |  |  |
| Toll | 2 | 1 | Toll, putative (XP00242097) [*Pediculus humanus*] 7e-62  Toll protein (ADK55066) [*Penaeus monodon*] 2e-56  Toll-6 (NP524081) [*Drosophila melanogaster*] 5e-41 |
| Pelle/NF-kappa-B essential modulator |  | 1 | Pelle (NP476971) [*Drosophila melanogaster*] 1e-12  Interleukin-1 receptor-associated kinase 4 (EDL04264)isoform CRA a [*Mus musculus*] 3e-13 |
| **Hedgehog** |  |  |  |
| Gli/Cubicus interruptus (CuI) | 1 | 4 | Gli-Kruppel type zinc finger protein (EFX72414) [Daphnia pulex] 4e-73  Gli-Krupple related protein (AAA59467) [*Homo sapiens*] 4e-75 |
| Patched homolog 1 (Ptch1) | 1 | 2 | Patched (NP523661) [*Drosophila melanogaster*] 6e-44  Patched domain-containing protein 3 (NP083325) [*Mus musculus*] 1e-15 |
| Megalin/ Low density lipoprotein receptor-related protein (Lrp2) |  | 4 | Megalin (NP001096924) [*Drosophila melanogaster*] 2e-51 |
| **Ecdysone** |  |  |  |
| Ecdysone-induced protein 63E | 1 |  | cdc2-related kinase (BAA21484) [*Bombyx mori*] 4e-29 |
| Phantom | 2 |  | Ecdysone biosynthesis protein (EFX88041) [*Daphnia pulex*] 9e-33  Phantom (NP 573319) [*Drosophila melanogaster*] 3e-15 |
| Shadow | 1 |  | Cytochrome P450 (XP 002411841) [*Ixodes scapularis*] 2e-17 |
| Disembodied | 1 |  | Cytochrome P450 3A41 (NP059092) [*Mus musculus*] 3e-37 |
| Ecdysoneless | 1 |  | Ecdysoneless (NP 647707)[Drosophila melanogaster] 1e-6 |
| Ecdysone-induced protein 75B |  | 1 | Ecdysone-induced protein 75B, isoform D (NP 730323) [*Drosophila melanogaster*] 2e-14 |
| **RTK** |  |  |  |
| Ras oncogene at  85D (Ras85D) | 2 |  | Ras-related nuclear protein (AAY96645) [*Marsupenaeus japonicas*] 1e-110  Ras oncogene at 85D (NP 476699) [*Drosophila melanogaster*] 5e-16 |
| Downstream of Raf1 (Dsor1) | 1 |  | Dsor1 (BAA02925) [*Drosophila melanogaster*] 6e-88 |
| **Germ plasm** |  |  |  |
| Vasa | 2 | 1 | Vasa (ACH92926) [*Parhyale hawaiensis*] 0  DEAD box ATP-dependent RNA helicase, putative (EEB18571) [*Pediculus humanus corporis*] 1e-170 |
| Nanos | 2 |  | Nanos (ABX80279) [*Parhyale hawaiensis*] 0 |
| Mago-nashi | 1 |  | Mago-nashi (ACJ47903) [*Penaeus monodon*] 3e-78  Mago nashi (NP476636) [*Drosophila melanogaster*] 7e-71 |
| **Hox genes** |  |  |  |
| Ultrabithorax | 2 |  | Ultrabithorax isoform II (ACT53742) [*Parhyale hawaiensis*] 2e-75 |
| Abdominal A( Abd-a)/ Hoxb7 | 1 |  | Abd-A (ABD16213) [*Strigamia maritime*] 9e-58  Abd-A (EFX86800) [*Daphina pulex*] 4e-52 |
| Sex combs reduced (Scr)/ Hoxa5 | 1 |  | Scr (NP 524248) [*Drosophila melanogaster*] 4e-44  Scr (NP 001037339) [*Bombyx mori* ] 8e-44 |
| Proboscipedia (pb)/ Hoxa2 | 1 | 1 | Proboscipedia, putative (EFX86812) [*Daphnia pulex*] 3e-14 |
| **Others** |  |  |  |
| Odd-skipped 2 | 3 |  | Odd-skipped 2 protein (ABK56707) [*Parhyale hawaiensis*] 4e-66 |
| Odd-paired | 1 |  | Transcription factor odd-paired, putative (EFX70006) [*Daphnia pulex*] 5e-32 |
| Shavenbaby |  | 1 | Shavenbaby (CAD23206) [*Drosophila melanogaster*] 1e-13 |
| Par-1/ MARK2 | 1 | 1 | Serine/threonine-protein kinase MARK2, putative (XP002431036) [*Pediculus humanus corporis*] 2e-28  Par-1, isoform S (NP001163210) [*Drosophila melanogaster*] 5e-27 |
| Maternal expression at 31B | 1 |  | Maternal expression at 31B, isoform A (NP523533) [*Drosophila melanogaster*] 4e-124 |
| Sox2/SoxNeuro | 2 |  | transcription factor Sox-2, putative (EEB17364) [*Pediculus humanus corporis*] 3e-39  SoxNeuro (EFA04654) [*Tribolium castaneum*] 3e-41 |
| Sox box protein 14 | 1 |  | Sox box protein 14, isoform A (NP599117) [*Drosophila melanogaster*] 3e-27 |
| Scribbled | 1 |  | Scribbled, isoform A (NP733155) [*Drosophila melanogaster*] 1e-48 |
